# Dietary Magnesium and Incident Frailty in Older People at Risk for Knee Osteoarthritis: An Eight-Year Longitudinal Study

**DOI:** 10.3390/nu9111253

**Published:** 2017-11-16

**Authors:** Nicola Veronese, Brendon Stubbs, Stefania Maggi, Maria Notarnicola, Mario Barbagallo, Joseph Firth, Ligia J. Dominguez, Maria Gabriella Caruso

**Affiliations:** 1 Ambulatory of Clinical Nutrition, Research Hospital, IRCCS “S. de Bellis”, Castellana Grotte, 70013 Bari, Italy; maria.notarnicola@irccsdebellis.it (M.N.); gabriella.caruso@irccsdebellis.it (M.G.C.); 2 Laboratory of Nutritional Biochemistry, National Institute of Gastroenterology-Research Hospital, IRCCS “S. de Bellis”, Castellana Grotte, 70013 Bari, Italy; 3 National Research Council, Neuroscience Institute, Aging Branch, Via Giustiniani, 2, 35128 Padua, Italy; stefania.maggi@in.cnr.it; 4 South London and Maudsley NHS Foundation Trust, Denmark Hill, London SE5 8AZ, UK; brendon.stubbs@kcl.ac.uk; 5 Faculty of Health, Social Care and Education, Anglia Ruskin University, Bishop Hall Lane, Chelmsford CM1 1SQ, UK; 6 Institute of Psychiatry, Psychology and Neuroscience (IoPPN) King’s College London, De Crespigny Park, London SE5 8AF, UK; 7 Geriatric Unit, Department of Internal Medicine and Geriatrics, University of Palermo, 90142 Palermo, Italy; mario.barbagallo@unipa.it (M.B.); ligia.dominguez@unipa.it (L.J.D.); 8 NICM Health Research Institute, University of Western Sydney, Sydney, NSW 2751, Australia; joefirth@gmail.com

**Keywords:** frailty, magnesium, older adults, Osteoarthritis Initiative

## Abstract

Inadequate magnesium (Mg) intake is associated with lower physical performance, but the relationship with frailty in older people is unclear. Therefore, we aimed to investigate whether higher dietary Mg intake is associated with a lower risk of frailty in a large cohort of North American individuals. Details regarding Mg intake were recorded through a food-frequency questionnaire (FFQ) and categorized as greater than/equal to Recommended Dietary Allowance (RDA) vs. lower. Frailty was defined using the Study of Osteoporotic Fractures index. Multivariable Cox’s regression analyses, calculating hazard ratios (HRs) with 95% confidence intervals (CIs), were undertaken by sex. In total, 4421 individuals with knee osteoarthritis or who were at high risk without frailty at baseline (mean age: 61.3, females = 58.0%) were followed for 8 years. After adjusting for 11 potential baseline confounders, reaching the RDA for Mg lowered risk of frailty among men (total *n* = 1857, HR = 0.51; 95% CI: 0.26–0.93), whilst no significant associations were found in women (total *n* = 2564). Each 100 mg of dietary Mg intake at baseline corresponded to a 22% reduction in men (HR = 0.78; 95% CI: 0.62–0.97; *p* = 0.03), but not in women (HR = 1.05; 95% CI: 0.89–1.23). In conclusion, higher dietary Mg intake appears to reduce the risk of frailty in men, but not in women.

## 1. Introduction

Frailty is a clinical syndrome that identifies older subjects with increased vulnerability to stress [1]. Frailty is a common condition, with an estimated prevalence of about 10% among community-dwelling older people [2], with even higher numbers reported among older people in long-term care [3]. Frailty has been associated with an increased risk of several deleterious outcomes, including disability, falls, hospitalization, institutionalization, and death [4], but recent literature suggests that frailty might also be associated with a higher risk of metabolic [5], psychiatric [6], and cardiovascular disease [7]. It is widely assumed that early interventions for frailty, particularly in the pre-frailty state, might improve quality of life, reducing adverse outcomes and associated costs of care [8].

The pathophysiology of frailty is complex and multi-factorial. However, nutrition is an important modifiable factor in its onset and therefore a clear target for intervention [9]. It has been reported that frailty is associated with lower levels of several micronutrients, including vitamins and minerals, such as magnesium (Mg) [10]. Mg has a pivotal role in muscle function and it is essential for energy metabolism, transmembrane transport, and muscle relaxation and contraction [11]. In humans, lower Mg levels seem to be associated with a significant lower muscular function [12,13]. This effect could be due to several mechanisms, including higher oxidative stress [14], and inflammatory [15], and insulin-resistance [16] pathways. However, to the best of our knowledge, no longitudinal study has reported a possible association between Mg and the incidence of frailty. Understanding if low Mg is associated with higher risk of frailty is of importance, since the deficiency of Mg is easily reversible with appropriate dietary suggestions or supplementation [17].

Given this background, the aim of the present study was to investigate the association between dietary Mg intake at baseline and the onset of frailty in a large cohort of North American people over a follow-up period of 8 years. Since previous literature has reported that Mg is effective in improving physical function, we hypothesized that subjects with a higher Mg intake would be at lower risk of developing frailty over time.

## 2. Materials and Methods

### 2.1. Data Source and Subjects

Data were obtained from the Osteoarthritis Initiative (OAI) database, available for public access at http://www.oai.ucsf.edu/ [18]. The specific datasets utilized were registered during the baseline and screening evaluations (V00) and each database reported data on frailty until 96 months from baseline (V10). Patients at high risk of knee osteoarthritis (OA) were recruited at four clinical centers (Baltimore, MD, USA; Pittsburgh, PA, USA; Pawtucket, RI, USA; and Columbus, OH, USA) between February 2004 and May 2006.

All the participants provided written informed consent. The OAI study protocol was approved by the institutional review board of the OAI Coordinating Center, University of California, San Francisco.

### 2.2. Baseline Dietary Magnesium Intake (Exposure)

Dietary Mg intake was obtained through a food frequency questionnaire recorded during baseline visit of the OAI [19]. Since this questionnaire included data on Mg supplementation, this intake was also considered. Then, the population was categorized in two main groups (consumption equal to/greater than or less than the corresponding Recommended Dietary Allowance, RDA) using the cut-offs of 420 mg for men and 320 mg for women, as suggested by the Institute of Medicine (IOM) [20].

### 2.3. Incident Frailty (Outcome)

The study’s outcome of interest was incident frailty. Frailty was assessed as outcome at wave 1 (12 months), 3 (24 months), 5 (36 months), 6 (48 months), 8 (72 months) and 10 (96 months). In agreement with the Study of Osteoporotic Fracture (SOF) index and other studies performed through the OAI [21,22,23,24,25], frailty was defined as the presence of at least 2 of the following 3 criteria: (1) weight loss ≥5% between baseline and any follow-up examination (since no information regarding weight changes were available at baseline, we considered those with a body mass index (BMI) of <20 kg/m^2^ to fulfill this criteria only for baseline); (2) the inability to rise from a chair five times without arm support (poor chair stand time); and (3) poor physical performance based on Question 10 of the SF12 questionnaire, i.e., responding to the question “in the past 4 weeks, did you feel full of energy?” with either “A little of the time” or “none of the time”.

### 2.4. Covariates

Other than the number frailty criteria at baseline (categorized as one vs. none), we identified several potential confounders at baseline in the association between Mg supplementation and frailty, including: daily energy intake (in kcal/day); BMI (measured by a trained nurse); physical activity evaluated using the Physical Activity Scale for the Elderly (PASE) [26]; depressive symptoms evaluated through the Center for Epidemiologic Studies Depression Scale [27]; race (whites vs. others); smoking habits (current/previous vs. never smokers); educational level (college degree vs. below) and yearly income (< or ≥US$50,000 or missing data). Validated general health measures of self-reported comorbidities were assessed using the modified Charlson co-morbidity score [28].

### 2.5. Statistical Analyses

Since the interaction sex by dietary Mg intake at baseline with incident frailty as outcome was significant (*p* < 0.0001), the data are reported by sex.

Normal distributions of continuous variables were tested using the Kolmogorov–Smirnov test. Data are shown as means ± standard deviations (SD) for quantitative measures, and percentages for all discrete variables. The difference in baseline characteristics between those reaching the corresponding RDA or not was tested by the independent *t*-test for continuous variables and the chi-square test for categorical ones.

Cox’s regression analysis was used to assess the strength of the association between Mg intake at baseline and the onset of frailty during follow-up. Significantly different factors with respect to those reaching or not reaching the corresponding RDA in at least one sex or factors significantly associated with incident frailty at univariate analysis in at least one sex were included. Multi-collinearity among covariates was assessed using the variance inflation factor (VIF), with a score of 2 leading to the exclusion of a variable, but no parameter was excluded for this reason.

The basic model was adjusted for age. In the fully-adjusted model the variables used were: age (as a continuous variable); race (whites vs. others); BMI (as a continuous variable); education (college degree vs. below); smoking habits (current and previous smokers vs. others); yearly income (categorized as ≥ or <US$50,000 or missing data); Physical Activity Scale for Elderly score (as a continuous variable); Charlson co-morbidity index (as a continuous variable); Center for Epidemiologic Studies Depression Scale (as a continuous variable); number of frailty indexes at baseline (one vs. none); and total energy intake (in kcal/day). The proportional hazard assumption was verified considering Schoenfeld’s residuals of the covariates [29]. Cox’s regression analysis estimates were reported as hazard ratios (HRs) with 95% confidence intervals (CIs). A similar analysis was run using Mg intake as continuous variable, with increases in 100 mg.

Several sensitivity analyses were conducted evaluating the interaction between dietary Mg at baseline and selected factors (i.e., age below or greater than/equal to 65 years, overweight/obese vs. normal weight, yearly income, sex, race, education, smoking habits, yearly income, and number of frailty indexes at baseline categorized as one vs. none) in the association with frailty, but only sex emerged as moderator of our findings (*p* < 0.05 for the interaction).

All the analyses were performed using SPSS 17.0 for Windows (SPSS Inc., Chicago, IL, USA). All statistical tests were two-tailed and statistical significance was assumed for a *p*-value < 0.05.

## 3. Results

### 3.1. Sample Selection

The OAI dataset initially included a total of 4796 American participants. Among them, 22 participants were excluded since they were already frail at baseline and another 353 were excluded since they did not provide data regarding frailty during follow-up, resulting in a final sample of 4421 participants.

### 3.2. Descriptive Characteristics

Of the 4421 participants included, 1857 were males and 2564 were females, with a mean age of 61.3 years (±9.2 years; range: 45–79). Only 819 (233 men and 586 women; =18.5% of the baseline population; 12.5 vs. 22.9% in men and women, *p* < 0.0001) reached the corresponding RDA, even though 2991 participants (=67.7%) took Mg supplementation.

Table 1 shows the participants’ characteristics by their Mg intake at baseline categorized as reaching the corresponding RDA or not, by sexes. In both men and women, people reporting a dietary Mg intake equal to/greater than the corresponding RDA were more physically active (*p* = 0.03 for both comparisons), whilst no significant differences emerged in terms of co-morbidities or depressive symptoms (Table 1). Finally, no significant differences emerged in terms of people reporting poor physical performance, poor chair stand time, or weight loss between people reaching the Mg RDA or not in both sexes (Table 1).

### 3.3. Dietary Magnesium Intake and Incident Frailty

During the 8-year follow-up, 362 subjects (120 men and 242 women; 8.2% of the baseline population) developed frailty corresponding to a global incidence rate of 12 (95% CI: 10–13)/1000 persons-year. Among the singular criteria, the most frequent criterion during follow-up was poor chair stand time (*n* = 780, 17.6%), followed by poor physical performance (*n* = 503, 11.4%), and weight loss (*n* = 19, 0.4%).

**Figure 1:**
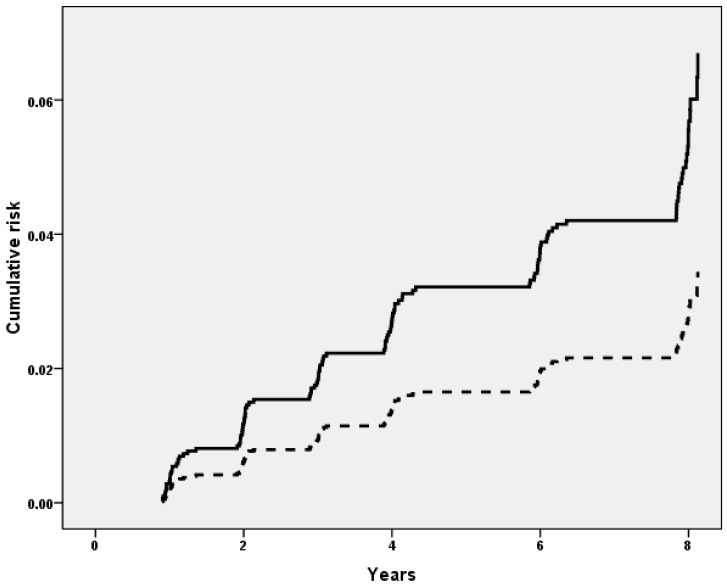
Risk of incident frailty in men by Recommended Dietary Allowance at baseline. Legend: the continuous line represents men not reaching the corresponding RDA at baseline; and the dashed line represents men reaching the RDA.

Table 2 shows the association between dietary Mg intake at baseline and incident frailty at follow-up. In men, the incidence of frailty among those reaching the corresponding RDA was significantly lower than for those not reaching this cut-off (6, 95% CI: 3–12 vs. 10, 95% CI: 8–12/1000 persons-year; *p* = 0.03), whilst no significant differences emerged for women (14, 95% CI: 12–16 vs. 16, 95% CI: 12–20/1000 persons-year; *p* = 0.36). After adjusting for 11 potential confounders at baseline, men reaching the RDA had a significant lower risk of frailty of about 49% (HR = 0.51; 95% CI: 0.26–0.93; *p* = 0.03) (Table 2; Figure 1), whilst no significant differences emerged for women (HR = 1.02; 95% CI: 0.71–1.46; *p* = 0.92) or in the sample as whole (HR = 0.88; 95% CI: 0.65–1.20; *p* = 0.43).

Modelling dietary Mg intake as a continuous variable and after adjusting for the potential confounders mentioned before, each increase in 100 mg of dietary Mg intake at baseline corresponded to a significant reduction in frailty incidence of 22% in men (HR = 0.78; 95% CI: 0.62–0.97; *p* = 0.03), but not in women (HR = 1.05; 95% CI: 0.89–1.23; *p* = 0.59) or in the population as whole (HR = 0.95; 95% CI: 0.83–1.08; *p* = 0.40).

## 4. Discussion

To the best of our knowledge, the current study is the first to investigate the relationship between dietary and supplementary Mg intake and incident frailty. In summary, the present study involving a large number of persons living in North America found that higher baseline Mg intake is associated with a lower risk of frailty in men, but not in women, after 8 years of follow-up. The risk of frailty in men reaching the RDA was almost halved and each increase in 100 mg of Mg intake decreased the risk of frailty by about 22%.

At baseline, no significant differences emerged between people reaching or not the RDA in both sexes in terms of potential risk factors for frailty, such as age, co-morbidities, or the presence of any frailty item. However, people reporting higher Mg intake were more physically active (as shown by higher PASE scores) and this probably played a role in the lower development of frailty in subjects reaching the corresponding RDA for Mg. However, it should be noted that our analyses were adjusted for this factor, suggesting that appropriate Mg intake can have an additional effect on frailty risk.

Another important consideration is the large number of people not reaching an appropriate intake of Mg. Our data, even if collected in a special population (i.e., people with knee OA or at higher risk of this condition), are similar to those collected in other American surveys. Using data from the NHANES (National Health and Nutrition Examination Survey) study, average intakes of Mg from food alone were higher among users of dietary supplements (350 mg for men and 267 mg for women) than among non-users (268 mg for men and 234 for women), but were still broadly insufficient [30]. Other surveys reported that almost half of the North American population consumed less than the required amount of magnesium from food in 2005–2006, and the figure was down from 56% in 2001–2002 [31]. Collectively, these data suggest that, despite the widespread use of Mg supplements, dietary Mg intake is insufficient in the American population, likely due to low consumption of green leafy vegetables, legumes, nuts, seeds, and whole grains [32], good sources of dietary Mg.

It is widely known that poor nutritional status is associated with the onset of frailty [9,33]. However, the possible association between Mg intake was poorly explored and the data available mainly derived from the literature regarding Mg and physical function [34]. Altogether, the previous findings available showed that higher Mg levels are associated with better physical function [11,12,13]. However, the design of previous studies (i.e., either cross-sectional or small randomized controlled trials) limits the strength of their findings. Thus, the findings from this longitudinal study involving more than 4000 participants substantially advance our understanding on this topic.

A point of importance is that higher Mg supplementation is associated with lower incidence of frailty in men, but not in women. We can hypothesize that since frailty has different prevalence and incidence between men and women [35,36], the risk factors for frailty (including nutritional factors) are probably different. Unfortunately, there was inadequate data to confirm which specific risk factors may differ between the sexes. However, with regards to Mg, it is known that women have lower Mg intake than men, although it is still debated if a different grade of absorption exists between sexes [37], leading to a different effect on muscle function. Thus, more studies are needed to better identify and understand the different roles of Mg in determining frailty between sexes.

The present findings should be considered within the limitations of the study. First, we had no data about Mg status, except for dietary Mg. Even if the best method for the assessment of Mg status is still equivocal [38,39], it would be interesting to assess if other parameters of Mg status can lead to different results. Second, we used a slightly different definition of frailty at baseline with respect to the one used at the follow-up, particularly with respect to weight loss. Unfortunately, no data regarding weight changes were available in the OAI at baseline and it is possible that some frail individuals at baseline were included in our analysis. Third, the participants of the OAI may not be representative of the general population, due to the inclusion/exclusion criteria of this study. Fourth, the inherent changes of Mg during time were not recorded and, consequently, it is not possible to assess the role of dietary Mg intake over time in the onset of frailty. Finally, the data regarding co-morbidities were self-reported and this can introduce another bias in our results.

## 5. Conclusions

In conclusions, our data suggests that higher dietary Mg intake is associated with a lower risk of frailty in men, but not in women, suggesting important sex differences in this potential relationship. Since low Mg intake is prevalent in the North American population, other studies are needed to see if Mg supplementation could be associated with lower risk of frailty in older people.

## Figures and Tables

**Table 1:** Characteristics of the participants classified according to their baseline dietary magnesium intake.

	Men			Women		
	Greater Than/Equal to RDA (*n* = 233)	Less than RDA (*n* = 1624)	*p*-Value ^a^	Greater Than/Equal to RDA (*n* = 586)	Less than RDA (*n* = 1978)	*p*-Value ^a^
**General characteristics**						
Age (years)	61.5 (9.0)	60.9 (9.5)	0.37	62.0 (8.8)	61.4 (9.0)	0.13
Energy intake (kcal/day)	2313 (701)	1488 (523)	<0.0001	1760 (553)	1143 (376)	<0.0001
PASE (points)	187 (96)	174 (86)	0.03	156 (80)	149 (75)	0.03
White race (*n*, %)	193 (82.8)	1388 (85.6)	0.28	456 (77.9)	1516 (76.6)	0.54
Smoking (previous/current) (*n*, %)	116 (50.2)	805 (49.8)	0.94	262 (44.9)	894 (45.4)	0.85
Graduate degree (*n*, %)	87 (37.3)	596 (36.8)	0.89	146 (24.9)	517 (26.2)	0.59
Yearly income (>US$50,000)	79 (33.9)	478 (29.4)	0.17	292 (50.2)	953 (48.2)	0.40
**Medical conditions**						
BMI (kg/m^2^)	28.2 (4.2)	28.9 (4.1)	0.08	28.5 (5.1)	28.6 (5.3)	0.67
CES-D (points)	6.3 (6.7)	5.9 (6.4)	0.35	6.9 (6.9)	7.0 (7.2)	0.61
Charlson co-morbidity index (points)	0.5 (1.1)	0.4 (0.9)	0.22	0.4 (0.8)	0.4 (0.8)	0.94
**Frailty items**						
Poor physical performance (*n*, %)	25 (10.8)	146 (9.0)	0.40	68 (11.6)	251 (12.7)	0.52
Poor chair stand time (*n*, %)	2 (0.9)	10 (0.6)	0.66	7 (1.2)	13 (0.7)	0.19
Weight loss (*n*, %)	3 (1.3)	11 (0.7)	0.40	23 (3.9)	62 (3.1)	0.36

Notes: The data are presented as means (with standard deviations) for continuous variables and numbers (with percentages) for the first and the fourth quartiles. ^a^ *p* values were calculated using the independent *t*-test for continuous variables and the chi-square test for categorical parameters. Magnesium RDAs were categorized using the cut-offs of 420 mg for men and 320 for women. Abbreviations: CES-D: Center for Epidemiologic Studies Depression Scale; PASE: Physical Activity Scale for the Elderly; BMI: body mass index; RDA: Recommended Dietary Allowance.

**Table 2:** Association between dietary magnesium intake and incidence of frailty.

	Incidence (95% CI) for 1000 Persons-Year	Basic Adjusted ^a^ HR (95% CI)	*p* Value	Fully Adjusted ^b^ HR (95% CI)	*p* Value
**Men**					
Greater than the RDA	10 (8–12)	1 (reference)		1 (reference)	
Greater than/equal to the RDA	6 (3–12)	0.70 (0.38–0.95)	0.03	0.51 (0.26–0.93)	0.03
**Women**					
Less than the RDA	14 (12–16)	1 (reference)		1 (reference)	
Greater than/equal to the RDA	16 (12–20)	1.13 (0.84–1.51)	0.43	1.02 (0.71–1.46)	0.92

Notes: All the data are presented as hazard ratios (HRs) with their 95% confidence intervals. ^a^ The basic adjusted model included as a covariate only age. ^b^ The fully-adjusted model included as covariates: age (as continuous); race (whites vs. others); body mass index (as continuous); education (degree vs. others); smoking habits (current and previous vs. others); yearly income (categorized as ≥ or <US$50,000 and missing data); Physical Activity Scale for Elderly score (as a continuous variable); Charlson co-morbidity index; CES-D: Center for Epidemiologic Studies Depression Scale; number of frailty indexes at baseline (one vs. none); and total energy intake (kcal/day). Abbreviations: CI: confidence interval; HR: hazard ratio; RDA: Recommended Dietary Allowance.
